# Establishment and Initial Testing of a Medium-Sized, Surgically Feasible Animal Model for Brucellar Spondylodiscitis: A Preliminary Study

**DOI:** 10.1155/2019/7368627

**Published:** 2019-09-30

**Authors:** Xiaoyu Cai, Tao Xu, Chuanhui Xun, Yakefu Abulizi, Qian Liu, Weibin Sheng, Zhihua Han, Liang Gao, Maierdan Maimaiti

**Affiliations:** ^1^Department of Spine Surgery, First Affiliated Hospital of Xinjiang Medical University, Urumqi, China; ^2^Center of Experimental Orthopaedics, Saarland University Medical Center, Homburg, Saarland, Germany; ^3^Sino Euro Orthopaedics Network, Homburg, Saarland, Germany; ^4^Department of Pathology, Basic Medical College, Xinjiang Medical University, Urumqi, China; ^5^Frankfurt Initiative for Regenerative Medicine, Experimental Orthopaedics and Trauma Surgery, J. W. Goethe University, Frankfurt, Germany

## Abstract

Brucellar spondylodiscitis, the most prevalent and significant osteoarticular presentation of human Brucellosis, is difficult to diagnose and usually yields irreversible neurologic deficits and spinal deformities. However, no animal models of Brucellar spondylodiscitis exist, allowing for preclinical investigations. The present study investigated whether intraosseous injection of attenuated *Brucella melitensis* vaccine into rabbits' lumbar vertebrae imitates the radiographic and histopathological characteristics of human Brucellar spondylodiscitis. Radiographic and histopathological analyses at 8 weeks postoperatively revealed radiographic changes within vertebral bodies and intervertebral discs, abscesses formation within the paravertebral soft tissue, and typical prominent inflammation response without caseous necrosis, which were largely comparable to human Brucellar spondylodiscitis. Such a medium-sized, surgically feasible rabbit model provides a promising *in vivo* setting for further preclinical investigation of Brucellar spondylodiscitis.

## 1. Introduction

Brucellosis is one of the most common and dangerous zoonoses caused by the Brucella bacteria [1, 2]. Human Brucellosis has become a public health concern with over 500,000 new cases annually and remains an enormous burden in both Asia and Africa [3]. As the most prevalent and important osteoarticular presentation of human Brucellosis, Brucellar spondylodiscitis has a variable course with unspecific symptoms and long latency between the onset of symptoms and the appearance of the radiologic changes, hindering an early intervention to prevent irreversible neurologic deficits and spinal deformities [4, 5]. Currently, no vaccines for human brucellosis exist due to incomplete understanding of Brucellar spondylodiscitis, and therefore, the disability and mortality rates remain considerably high in Brucellar spondylodiscitis patients [6].

Animal models exhibiting symptoms comparable to those in patients are essential for the translation of preclinical findings/outcomes from bench to bedside [7]. For translational research using small animal models (e.g., rodents), the power to predict clinical efficacy is in dispute due to their weakness in faithfully mirroring the extremely complex process of human disease and successfully translating preclinical findings to humans [8]. Previous tuberculosis research using larger animal models underlined the advantages of rabbits with a cell-mediated immune response and a delayed-hypersensitivity response similar to humans, compared with other species (e.g., guinea pigs and dogs) [9]. Also, rabbits are easily acquired and share significantly morphological and structural similarity to human spine [10], offering a promising candidate to establish such a disease model of Brucellar spondylodiscitis [11, 12]. Moreover, their relatively large body size allows easier surgical approaches and makes them largely convenient for *in vivo* investigations [13]. However, due to their size, high-cost, large space requirements and lack of immunological reagents, the rabbits currently have only been utilized to test *Brucella* toxicity [14]. Yet, to our very best knowledge, a Brucellar spondylodiscitis model in such a medium-sized animal with a possible high translational efficiency has not been established and validated to date.

In the present study, we investigated whether intraosseous injection of attenuated *Brucella melitensis* vaccine into rabbits' lumbar vertebrae imitates the radiographic and histopathological characteristics of human Brucellar spondylodiscitis.

## 2. Methods

### 2.1. Study Design

**Figure 1:**
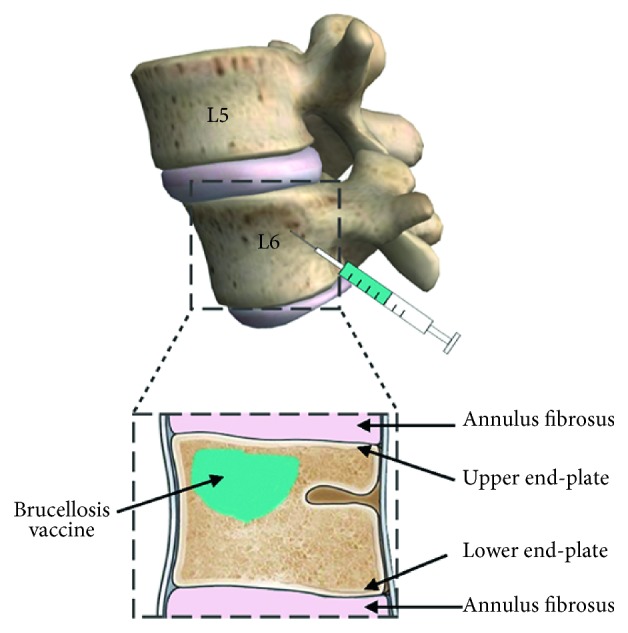
Schematic of the experimental challenge injection with *Brucella melitensis* into the 6th lumbar vertebrae (L6) of rabbits.

Standardized bony canals were drilled with a Kirschner wire below the upper end-plate of the 6th lumbar vertebrae (L6) into the vertebral body of rabbits. Attenuated *Brucella melitensis* vaccine and normal saline were injected into the canals for the experimental and sham surgery groups, respectively (Figure 1). Rabbits receiving no surgery served as normal controls. Animal status and treatment outcomes were evaluated by *in vivo* radiographic analyses, including plain radiography, computed tomography (CT) reconstruction, and magnetic resonance imaging (MRI), at both 4 and 8 weeks postoperatively. After the radiographic assessments at 8 weeks postoperatively, all animals were sacrificed, and the targeted vertebral and paravertebral structures were harvested for further macroscopic and histological analyses [15].

### 2.2. Animal Experiments

All animal experiments were conducted in agreement with the national legislation on protection of animals and the National Institutes of Health (NIH) Guidelines for the Care and Use of Laboratory Animals (NIH Publication 85–23, Rev 1985) and were approved by the Animal Ethics Committee of Xinjiang Medical University, Urumqi, China (approval number: SCKX XIN 2011-0001). The experiments were performed in a biosafety level 3 laboratory of Yili Vocational and Technical College and the animal laboratory of Xinjiang Medical University.

Thirty-six mature, healthy New Zealand white rabbits (mixed gender; mean age, 3 months; body weight, 2.5–3.0 kg) received water ad libitum and were fed a standard diet. A veterinarian continuously monitored all animals. Animals were randomly allocated into three groups: experimental group (12 rabbits), sham surgery group (12), and normal control group (12).

For preoperative preparation, animals were given small amounts of water and fasted for 12 hours. After placed at left lateral recumbent position, the skin at the incision site was disinfected once with 2% iodine tincture and twice with 75% alcohol, and animals were injected with 2% pentobarbital sodium (20 mg/kg, Tianjin Jinyao Pharmaceutical Co., Ltd., Tianjin, China) through the posterior auricular vein. A 6 cm long longitudinal incision was made from the end of the 12th rib to the anterior superior iliac spine. After the exposure of the 12th rib and the 5th (L5) and 6th (L6) lumbar vertebral segments, a Kirschner wire of 3 mm in diameter was drilled at 5 mm below the upper end-plate of 6th lumbar vertebrae down to a depth of 5 mm [16]. The suspension of the live attenuated *Brucella melitensis* vaccine strain M5-90 (0.1 ml, 3 × 10^8^ CFU/ml, Xinjiang Tiankang Pharmaceutical Co., Ltd., Urumqi, China) was slowly injected into the gelatin sponge (Nanjing Jinling Pharmaceutical Co., Ltd., Nanjing, China), and the bony canal was sealed with 0.1 ml bone wax.

In the sham surgery group, animals received the aforementioned procedures, and 0.1 ml of saline containing liquid culture medium (modified Sauton medium, self-made) was slowly injected into the lesions. In the normal control group, 12 rabbits did not receive any treatment. Wounds were closed standardizedly in layers. Animals were caged individually in the same environment and separated from each other, and animal rooms were disinfected by ultraviolet radiation for 1 hour every day. Animals were allowed immediate full weight-bearing, and they returned to normal activities.

### 2.3. *In Vivo* Radiographic Analyses

*In vivo* plain radiography (Computed Radiography Digital Imaging System, Neu-Alpine, China), computed tomography (CT) (Brilliance 64-slice CT scanner, Siemens, Germany), and magnetic resonance imaging (MRI) (MAGNETOM Symphony 1.5 T MRI system, Siemens, Germany) analyses were performed to observe the targeted vertebral body and intervertebral disc at 4 and 8 weeks postoperatively under the general anesthesia. Specifically, the MRI findings were classified into five types, such as discitis type, spondylitis type, paraspinal/psoas abscess type, appendicitis type, and compound type, with a previously reported classification system (Table 1) [17].

### 2.4. Macroscopic Observation

After the radiographic analyses at 8 weeks postoperatively, animals were sacrificed, and the L6 vertebrae and paravertebral structures were harvested for the macroscopic observation. Data were collected including the formation of bone sequestrum, paravertebral or psoas abscess, intervertebral space narrowing, and thoracic and abdominal cavity involvement [18].

### 2.5. Histological Analysis

Samples biopsied from the affected intervertebral disc, upper and lower end-plates, paravertebral soft tissue, psoas, and granulation tissue (if available) were processed using 10% formalin, graded alcohols, xylenes, and finally paraffin. Histopathology slices (5 *μ*m thick) were cut using a standard microtome and dried in a slide warming oven overnight and later stained with hematoxylin and eosin [19, 20]. Special attentions were paid to histomorphological patterns, including chemotaxis of inflammatory cells, emerging epithelioid and multinucleated giant cells, and formation of proliferative nodule [21].

## 3. Results

### 3.1. Postoperative Survival and Complications

In the experimental group, 1 rabbit was eliminated due to incision infections at 4 weeks postoperatively. Only 1 rabbit died within 8 weeks after the challenge injection without further necropsy. The success, death, and exclusion rates were 83.4%, 8.3%, and 8.3%, respectively. All survival rabbits presented signs of emaciation and decreased activity. No noticeable abnormalities of body shape were observed in either died or eliminated rabbits. In the sham surgery group, 1 rabbit died on the day of surgery. In the normal control group, all 12 rabbits survived over 8 weeks postoperatively with normal activities. The range of postoperative weight loss of the animals was 0.2–0.7 kg.

### 3.2. *In Vivo* Radiographic Analyses

**Figure 2:**
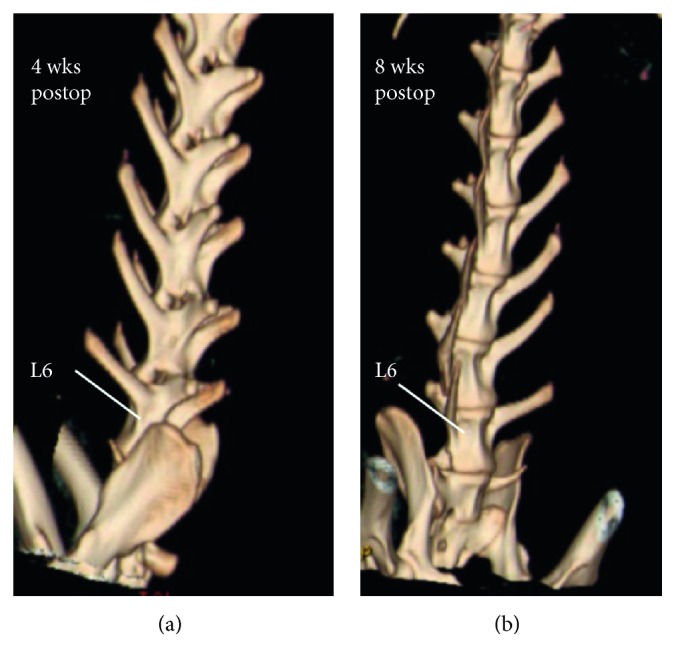
Representative 3D reconstructed CT images indicating no bony destruction of the vertebral body and calcification of the paravertebral soft tissue at 4 and 8 weeks postop, respectively.

**Figure 3:**
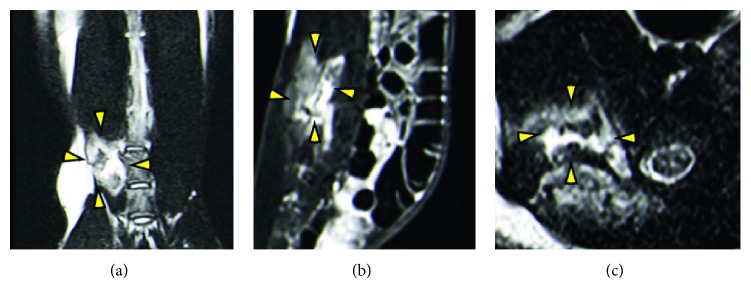
Representative T2WI MRI images in coronal (a), sagittal (b), and transverse (c) orientations of a rabbit with a type IV (compound type) Brucellar spondylodiscitis at 8 weeks postoperatively. Isosignal or slight hyperintense signal was observed within the L5 and L6 vertebral bodies and intervertebral discs and hyperintense signal within the paravertebral soft tissue. Yellow arrowheads designate the margin of a paravertebral abscess.

In the experimental group, both plain radiography and 3D CT reconstruction showed no evident intervertebral space narrowing, vertebral body destruction, and vertebral height loss (Figure 2). The T2 weighted image (T2WI) at 8 weeks postoperatively observed isosignal or slight hyperintense signal within the L5 and L6 vertebral bodies and hypersignal intense within the paravertebral soft tissue (Figure 3). According to the modified MRI classification system, all cases were further classified [17], in which the compound type was the most frequently type (70%) followed by the paraspinal abscess or psoas abscess type (20%) and discitis type (10%). None was classified as spondylitis or appendicitis type (Table 2). In both sham surgery and normal control groups, all radiographic scans did not identify noteworthy pathological changes within the vertebral and paravertebral structures.

### 3.3. Macroscopic Observation

In the experimental group, the incision healed well without sinus formation. After peeling the anterior vertebral muscle off the vertebral body, (1) the colour of the fibrous ring became darker and the intervertebral space became narrowed, (2) there was no obvious bone destruction in the adjacent upper and lower end-plates of the vertebral body, (3) the paravertebral soft tissue and psoas major muscle were oncotic without obvious abscess, (4) there were no necrotic substance and granulation tissue around the vertebral body, and (5) there was no difference between the thoracic cavity and other abdominal organs. In the sham surgery group, the diameter of the holes in the drilling site of vertebral body was reduced, and no abscess and vertebral bone destruction were observed. In the normal control group, the vertebral body, abdominal cavity, and thoracic organs were visually normal.

### 3.4. Histological Analyses

**Figure 4:**
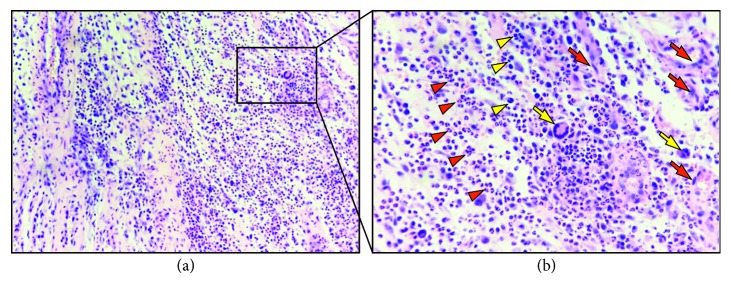
Hematoxylin and eosin staining features predominant lymphocyte and monocytes infiltration with sparsely distributed epithelioid cells and multinucleated giant cells in the experimental group. Yellow arrows indicate multinucleated giant cells and red arrows specify epithelioid cells. Yellow arrowheads designate lymphocytes and red arrowheads display monocytes (magnification: (a) ×40; (b) ×100).

In the experimental group, hematoxylin and eosin staining showed signs of infiltration of inflammatory cell with various spatial extents in all biopsied samples from the paravertebral structures at 8 weeks postoperatively. Overwhelmingly, the cellular infiltration was predominated with lymphocyte and monocytes with sparsely distributed clusters of epithelioid cells and multinucleated giant cells (Figure 4) at the high magnification field-of-view. Typical coagulation necrosis and bony sequestrum formation were not observed. In the sham surgery group, only a few inflammatory cells were detected without existing epithelioid cells.

## 4. Discussion

Brucellar spondylodiscitis, a common type human Brucellosis, has been increasing rapidly particularly in the underdeveloped regions [22], while no vaccines against human Brucellosis exist. Establishing appropriate animal models of Brucellar spondylodiscitis is an essential step for preclinical investigation and timely intervention of Brucellar spondylodiscitis. To the best of our knowledge, the present study is the first to establish and provide initial results of a medium-sized, intervention-friendly animal model of the Brucellar spondylodiscitis, which offers a promising *in vivo* setting for further preclinical investigation.

Rabbits are medium-sized animals and frequently used in spine research with various advantages compared with other animals [23–26]. Compared with smaller animals (e.g., mice or rats), rabbits maintain considerable morphological and structural similarity to human spine and their body size allows exposing during surgical approaches [10, 14, 27] and yield higher rates than rodents for successful translation of preclinical findings to humans [7, 8]. Similarly, compared with larger animals (e.g., pigs or sheep), radiographic analyses are largely convenient in rabbits particularly for *in vivo* investigations [13]. Furthermore, rabbits are also easily acquired and inexpensively fostered [10]. Therefore, establishing a rabbit model of Brucellar spondylodiscitis is technically feasible, translationally favourable, and economically applicable.

In the present study, young rabbits were used considering the significant variance of innate immune response between young and adult rabbits against foreign microbes. Previous studies of *Mycobacterium tuberculosis* showed that differential regulation of the innate immune response and related gene expression changes determined the long-term outcome of tuberculosis infection in rabbits and the disease progression was due to the continuous host response to bacterial products rather than the increasing number of viable bacilli [28–31]. Moreover, earlier studies of rabbit hemorrhagic disease caused by a calicivirus highlighted the significantly superior innate immune system in young rabbits (<4 weeks) over adult rabbits, which contributed to their distinct resistance/susceptibility to virus infections (90% adult rabbit but 0% young rabbits died within 3 days after the viral inoculation) [32–35]. Therefore, in the present study, we used the young rabbits to investigate the susceptibility of tested dose of *Brucella melitensis* and to establish a local restricted inflammation without systemic dissemination of the microbes. Additional studies with both young and adult rabbits are required to elucidate the possible confounding effect of the animal age to establish effectively such a rabbit model of Brucellar spondylodiscitis.

Live attenuated Brucella melitensis vaccine (strain M5-90) was selected to test in this animal model. The strain M5-90 is derived from virulent strain M28 isolated from sheep after serially passaging through chickens, treatment with acriflavine, and further passaging for 90 generations in chicken embryo fibroblasts. Vaccination with strain M5-90 is one of the important strategies that decreased the incidence of animal Brucellosis during 1970s to 1990s in China [36]. Moreover, to boost a localized inoculation into the vertebral body and minimize the vascular dissemination, a relatively large dose of vaccines was meticulously implanted within the superior zone of the anterior column of L6 vertebral body, where the direct vertebral body feeding capillaries scantily supplied [37].

To ensure an accurate localization of the bacterial inoculation [38], we performed the open surgery to expose the target vertebrae for intraosseous injection rather than the radiograph-guided percutaneous injection, which has been frequently applied in animal models of pyogenic spondylodiscitis [39]. Bierry et al. reported the rabbit model of pyogenic spondylodiscitis through the injecting of *Staphylococcus aureus* with a spinal needle (no. 22) into the L3/L4 and L5/L6 intervertebral discs under the fluoroscopic guidance [40]. Likewise, Wang and colleagues established the similar rabbit model of pyogenic spondylodiscitis by the fluoroscopy-guided needle injection of the methicillin-resistant *Staphylococcus aureus* into the L4/5 intervertebral space [20]. There are two other reasons for using the open surgery over the percutaneous approaches to establish such an intraosseous injection model. Firstly, the midportion of the vertebral body of rabbits is very narrow, compared with other species (e.g., dogs, rats, and mice) and mostly composed of the cortical bone [41]; therefore, an intraosseous injection is practically difficult to perform. Secondly, the relatively narrow intervertebral space is not allowed for a large dose injection [42]; therefore, the present intraosseous injection model following the open surgery allowed for further comparison of possible dosage-related effects in rabbits of Brucellar spondylodiscitis.

Radiographs augmented with MRI imaging are used as the main clinical screening methods for human Brucellar spondylodiscitis, especially with MRI due to its high sensitivity and specificity [43–45]. At 8 weeks postoperatively, our radiographic data showed that rabbits injected with live *Brucella* vaccines largely imitate the features of the acute stage of human Brucellar spondylodiscitis. Specially, T2WI displayed isosignal or slight hyperintense signals within the L5 and L6 vertebral bodies, intervertebral discs, swollen neighboring soft tissue, and paravertebral abscess, which are consistent with the MRI findings in acute phase Brucellar spondylodiscitis in humans [5, 45]. However, in contrast to earlier findings, both CT and MRI images at 4 and 8 weeks postoperatively identified no osseous end-plate erosion, vertebral body osteomyelitis, intradiscal gas (a unique finding in brucellosis), and signs of vascular dissemination into adjacent epidural space in rabbits, which are generally presented in the diffuse form of human Brucellar spondylodiscitis [44, 46]. A possible explanation for this might be the partially restricted susceptibility to *Brucella* infection in rabbits, which might be compensated with higher vaccine dosage or multiple injections [23].

Moreover, the pathological characteristics of Brucellar spondylodiscitis in rabbits were also highly consistent with those in acute stage of human Brucellar spondylodiscitis. In human Brucellar spondylodiscitis, microgranulomatous proliferation with histiocytes and without caseous necrosis is characteristic [38]. In rabbits, histopathological analyses identified massive inflammatory cell infiltration without evident bony erosions within the biopsied paravertebral structures, including lymphocytes, monocytes, and multinucleated giant cells, which also partially confirmed the previous reported osteoarthritic changes in genetic modified mice infected intraperitoneally with *Brucella melitensis* [47]. Consistent with our radiographic findings, no prominent bone and disc destruction were notified, which can be explained in part by the lacking of proteinase activity within *Brucella melitensis* to destroy the collagenous matrix and the induced osteoblastic activity to preserve the bony structure of vertebrae [48].

Several surgical pearls and pitfalls need to be highlighted during the animal model establishment. The key to establish this animal model is to implant the *Brucella* into the superior zone of the anterior column of L6 vertebral body; therefore, the insertion site, direction, and drill depth of the Kirschner wire should be properly monitored. Also, different patterns of level of the nerve root origin and adjacent vertebra in rabbits from humans need to be recognized to avoid iatrogenic nerve injuries [49].

This study holds several limitations. As a preliminary study, no comparative studies were performed with the different batches of bacterium and dosages of injection. Moreover, the most frequent clinical symptoms in human Brucellar spondylodiscitis are local pain and fever with latent phase of unknown duration [50, 51], which were practically difficult to monitor in such a rabbit model established by the local inoculation [52–54]. Also, considering risks of bacteria dissemination, blood test and bacteria culture were not accomplished in the present study; however, the available data of radiographic and histopathological analyses have already preliminarily tested the feasibility of such a rabbit model of Brucellar spondylodiscitis and offered valuable hints for further investigations.

## 5. Conclusion

The present study established and tested a medium-sized, surgically feasible animal model for Brucellar spondylodiscitis, providing a promising *in vivo* venue for further preclinical investigations.

## Figures and Tables

**Table 1:** Modified classification of Brucellar spondylodiscitis for MRI [17].

Classification	MRI characteristics
Discitis	Regional inflammation involving intervertebral disc
	Disc space narrowing
	Low signal on T1-weighted image mixing high signal on T2-weighted image
Spondylitis	Regional inflammation involving adjacent vertebrae
	Vertebrae diffuse marrow edema
	Homogeneous or uneven low signal on T1-weighted image of vertebrae
Paraspinal/psoas abscess	Regional inflammation involving paraspinal or psoas
	Paravertebral abscess
	Psoas abscess
Appendicitis	Regional inflammation involving appendicitis
	Low signal on T1-weighted image
	High signal on T2-weighted image
Compound	Endemic inflammation involving two or more parts of vertebral and paravertebral structures
	T1-weighted image reveals incomplete heterogeneous hypointensity
	T2-weighted image reveals hyperintensity

**Table 2:** Demographic classification of Brucellar spondylodiscitis according to the modified classification of the magnetic resonance imaging.

Groups	Postoperative outcome			Modified MRI classification				
	Success	Death	Exclusion	Discitis	Spondylitis	Paraspinal/psoas abscess	Appendicitis	Compound
Experimental	10/12	1/12	1/12	1/10	0/10	2/10	0/10	7/10
Sham surgery	0/12	1/12	0/12	0/11	0/11	0/11	0/11	0/11
Normal control	0/12	0/12	0/12	0/12	0/12	0/12	0/12	0/12

Success: successful establishment of the animal model confirmed by radiographic and histopathological evidences.

## Data Availability

The datasets of the present study are available from the corresponding authors on reasonable request.
